# How prevalent are symptoms and risk factors of pelvic inflammatory disease in a sexually conservative population

**DOI:** 10.1186/s12978-021-01155-2

**Published:** 2021-05-28

**Authors:** Oqba Al-kuran, Lama Al-Mehaisen, Hamza Alduraidi, Naser Al-Husban, Balqees Attarakih, Anas Sultan, Zeina Othman, Sanal AlShárat, Shoug AlHilali, Nadia Alkouz, Noura Alibrahim, Wafaa AlMusallam

**Affiliations:** 1 Obstetrics and Gynecology Department, School of Medicine, University of Jordan, Amman, Jordan; 2 Obstetrics and Gynecology Department, School of Medicine, Al-Balqa Applied University, Salt, Jordan; 3 Community Health Nursing Department, School of Nursing, The University of Jordan, Amman, Jordan; 4 School of Medicine, The University of Jordan, Amman, Jordan

**Keywords:** Pelvic inflammatory disease (PID), Women’s health, Infertility, Contraceptives, Chronic pelvic pain, Jordan

## Abstract

**Background:**

Pelvic inflammatory disease (PID) is the inflammation of the adnexa of the uterus, that mainly manifests in a subclinical/chronic context and goes largely underreported. However, it poses a major threat to women’s health, as it is responsible for infertility and ectopic pregnancies, as well as chronic pelvic pain. Previous studies in Jordan have not reported PID, attributed mainly to the social structure of the country which largely represents a sexually conservative population. Our study aims to report the clinical symptoms that point towards PID and investigate the major risk determinants for the Jordanian population, in a cross-sectional study, using our scoring system based only on clinical data and examination.

**Methods:**

One hundred sixty-eight consecutive adult women that came in the Outpatient Clinics of Gynaecological Department of the Jordan University Hospital were interviewed and their medical history and symptoms were registered and analysed. A Score for PID symptoms, we developed, was given to each woman. Results and correlations were then statistically tested.

**Results:**

Our study population consisted of relatively young women (37.7 ± 11) that had their first child at an average age of 24.1 (± 4.8) and a mean parity of 3.1 (± 2.2). Fifty-eight women (34.5%) reported having undergone at least one CS, while the mean PID Symptom Score was 3.3 (± 2.3). The women in our study exhibited 8 symptoms of PID, namely dysmenorrhea and vaginal discharge; being the commonest (45.2% and 44.6% respectively), in addition to chronic pelvic pain, pelvic heaviness, menorrhagia, dyspareunia, urinary symptoms, and smelly urine. They also reported history of 3 conditions that can be attributed to PID, that is infertility, preterm labour, and miscarriages.

**Conclusions:**

Our PID Scoring System seems to identify the risk factors of PID and predict well the PID likelihood. This score predicts that women with higher parity, who used contraceptives and underwent any invasive medical procedure are expected to score higher in the PID Symptom Score. Our data also suggest that PID should not be ruled out in the Jordanian population when symptoms are compatible to this diagnosis.

## Plain language summary

As a sexually conservative country, Jordan is thought to have a low prevalence of pelvic inflammatory disease. The prevalence of STD pathogens is very low, however many patients present symptoms of PID, so we randomly interviewed 168 healthy participants and investigated symptoms related to PID. Surprisingly the percentage of participants who had symptoms of PID was high, reaching up to 64% for some symptoms. We then created a PID symptom score; where every symptom gets one mark (1–11), and tested it for association against independent factors. As a result, it can be predicted that a woman with higher parity, who used contraceptives, and underwent E & C, D & C, HSG, or Hysteroscopy is expected to score higher in the PID Symptom Score.

This result draws the attention to PID incidence in similar conservative communities, and therefore further research is needed to confirm the prevalence of PID and identify the causative factors.

## Background

Pelvic inflammatory disease (PID) is the inflammation of the adnexa of the uterus, namely the uterus, the fallopian tubes, the ovaries, and the pelvis. It is caused by persistent pathogenic infections that permits the microorganisms to ascend from the initial infection point (the vagina and the endocervix) to the endometrium or beyond [1]. It presents a range of clinical manifestations from totally asymptomatic to endometritis, parametritis, tubo-ovarian abscess, salpingitis, oophoritis, pelvic peritonitis, perihepatitis (Fitz–Hugh–Curtis syndrome) and even ovarian carcinogenesis [2]. PID is the cause of about 30% of infertility cases and 50% of ectopic pregnancies, therefore it presents a significant public health and economic burden, for women in the reproductive age [3].

Despite its obvious importance in women’s health, the prevalence of PID is unclear because it is largely underreported, either because it is asymptomatic or with mild symptoms [4] or because of social and ethical constraints. Due to financial and technical difficulties, PID prevention programs based on pathogen screening are not available or reliable in many countries, thus the actual burden of PID may be even greater than anticipated [5]. A self-reporting USA survey, in 2013–2014, estimated the PID incidence to 4.4% [6], a slight decline from previous reports [7]. USA currently runs a preventive program against chlamydia and gonorrhoea infection in adolescents, to help prevent PID, but questions are raised on whether youngsters might be willing to participate [8].

The identification of the pathogen responsible for PID is hampered by the imprecision in diagnosing PID, the difficulty in sampling the upper genital track [9], the frequent super-infection [10, 11] and the difficulty of identifying the pathogen [12]. Present data suggest that *N. gonorrhoeae*, *C. trachomatis* and/or *M. genitalium* are present in about 30% of PID cases [6, 13] and Bacterial Vaginosis-associated or urogenital pathobiontic bacteria (i.e. *S. agalactiae*, *Staphylococcus aureus* and Enterobacteriaceae) in about 70% of cases [14]. Some BV-associated organisms seem to be associated with PID, whereas others not [15].

While the incidence of PID is correlated strongly with the prevalence of sexually transmitted diseases, a fraction of the infections might be of endogenous origin. The use of intrauterine contraceptive devices and abortions procedures, even legal ones, contribute to the higher occurrence risk. A study in India linked the low socio-economic status, illiteracy, the use of intrauterine device, the number of sexual partners and the young age of marriage with the increased occurrence of PID [3], while in Nigeria PID is associated with polygamy practices [16]. Therefore, it is obvious that local traditions and practices may affect the actual prevalence and the reporting of the disease.

Jordan is an Islamic conservative country and sexuality is not encouraged outside wedlock. Therefore, it is not surprising that chlamydial infection is exceptionally low in Jordan, reaching 4.6% among symptomatic patients with urethritis, of both sexes [17]. An even older thesis estimated the *C. trachomatis* infection to 5.7% in men and 3.3% in women [18]. This is markedly lower than Western more liberal societies, where the chlamydial infection can reach as high as 39.3% in adolescent men and 11.1% in women in USA [19]. A USA report of the staggering 19.5% prevalence of PID in a cohort of adolescent females presenting to an urban emergency with abdominal or genitourinary complaints, shows the impact on western youth [20]. However, there is a rarity of PID reporting in Jordan, and an older seven-year report about ectopic pregnancy did not show any relevant PID aetiology [21]. In this study we attempt to investigate the amplitude of PID symptoms in Jordan’s women and to assess the relation of those symptoms to potential causative conditions, such as uterine instrumentation.

The aim of the present study is to provide the means of assessing PID occurrence in the Jordanian community. We developed a PID scoring system based on clinical symptoms and medical history, in order to facilitate the PID diagnosis, without any elaborate further testing. The purpose of this Scoring is to identify the high PID-risk women and provide an adequate triage system for women at risk in the Outpatient Clinics in Jordan. This triaging can help in designing an effective intervention that can minimize the occurence and developing a model that can act as a decision support system to better national health care.

## Methods

### Study design

This is a cross-sectional observational study conducted between August 2019 and March 2020. Patients included in this study were consecutive women that came to the Outpatient Clinics of Gynaecological Department of the Jordan University Hospital (JUH), Amman/Jordan, either as patients or visitors. This study focuses on the PID symptoms’ range and risk factors, in the Jordanian population.

### Study population

One hundred sixty-eight (168) non-pregnant married or previously married Jordanian women (i.e., sexually active), aged 18 years old and above were included in the present study.

### Inclusion criteria

The inclusion criteria required women to provide an informed written consent to participate in the study. They had to be 18 years old or above, confirmed non-pregnant, with no known history of previous genital infections. It is worth noting that, during this study, none of the participants reported having a prior PID diagnosis.

### Exclusion criteria

Given that our focus was the previously undiagnosed PID cases, we focus on healthy participants with no other health conditions, pregnancy included. Exclusion criteria were patients that refused to participate, underage women, pregnant women regardless of their age, as pregnancy can manifest some genital symptoms that can confound our PID findings. Moreover, patients that had a recent (less than 6 months) history of miscarriage or childbirth, were also excluded, as they are more likely to have PID already and could skew our results.

### Data collection and questionnaire

The data were collected using an electronic structured questionnaire filled by eight researchers, that participated in this study, during a short interview in the Outpatient Clinics. The questionnaire contained an informed consent form and medical history questions in three sections. The first section contained the demographic data, namely personal information such as name (optional), mobile phone (optional), date of birth and marriage age. Personal information was collected to be able to contact the women about a summary of this study’s results, if they were interested. In this first section, general obstetric details were recorded including parity and age at first birth. The second part contained the PID symptoms and complication including chronic pelvic pain, pelvic heaviness, dysmenorrhea, menorrhagia, dyspareunia, vaginal discharge, recurrent miscarriage, and infertility. The third portion contained some known predisposing factors such as lower pelvic surgeries including Caesarean Section (C/S) and Appendectomy, Dilation and Curettage (D&C), in vitro fertilization (IVF), Intracytoplasmic Sperm Injection (ICSI), use of intrauterine contraceptive device (IUCD), hysterosalpingography (HSG) and hysteroscopy.

The questionnaire was assessed and modified within the context of the outpatient clinic but has not been validated for the general population. This study is the first step for national validation and general implementation.

### Study tool-PID scoring system

The PID scoring was done by adding a mark for each positive response to the list of the known PID symptoms and complications, reported above. The score can range from 0 (no symptoms) to 11 (exhibiting all symptoms). Therefore, the higher score means that more symptoms are present for each participant.

Data were extracted to excel files and analysed statistically using SPSS software. Analysis included descriptive statistics (percentages and means) and correlation statistic (regression analysis).

### Ethical considerations

This study was approved by the Ethical Review Committee (ERC) of the Faculty of Medicine at the University of Jordan and the Institutional Review Board (IRB) at Jordan University Hospital.

Data collection was conducted inside a private clinic room and the women were asked for consent to participate in the research, before gathering their data. Confidentiality of the data was assured, the study aim was explained, and participants signed the consent. The names and other identifiers were covered and not divulged to researchers involved in the data analysis.

## Results

### Demographic data

Our study population consisted of 168 Jordanian women aged between 19 and 66 years, with a mean age of 37.7 years (SD = 11). Their age of marriage ranged from 12 and 40 years old, with a mean marriage age of 22.9 years old (SD = 5.1). The age upon their first childbirth ranged between 15 and 41 years old, with a mean age of 24.1 years old (SD = 4.8) and a mean parity of 3.1 (SD = 2.2). Table 1 summarizes the demographic data of the women studied. Most of the women (82.7%) were pre-menopausal and the majority were married (96.4%) at the time of data collection. Most women had less than 3 children, with the one child being more frequent, but this is expected, given the young age of the women participating.

**Table 1:** Demographic characteristics (N = 168)

Characteristic	No.	%
Marital status		
Married	162	96.4
Divorced	4	2.40
Widow	2	1.20
CS experiences		
0	110	65.50
1	23	13.70
2	18	10.70
3	8	4.80
4	6	3.60
> 4	6	3.60
Miscarriage		
0	96	57.10
1	44	26.20
2	16	9.50
3	7	4.20
> 4	5	3.00
Menopause		
Pre-	139	82.70
Post-	29	17.30

Fifty-eight women (34.5%) reported having undergone at least one CS. Of whom, 23 reported having CS only once, 18 women had CS twice, 8 women had CS three times, 6 women had CS four times, and 3 women reported having CS more than four times. Seventy-two women (42.9%) reported experiencing at least one miscarriage. Of whom, 44 women had miscarriage once, 16 women had miscarriage twice, 7 women had miscarriage three times, and only 5 women had more than three miscarriage experiences.

### Prevalence of PID symptoms

The women in our study exhibited 8 of the most common symptoms of PID, namely chronic pelvic pain, pelvic heaviness, dysmenorrhea, menorrhagia, dyspareunia, vaginal discharge, urinary symptoms, and smelly urine, and reported history of 3 conditions that can be attributed to PID, that is infertility, preterm labour, and miscarriages. Table 2 shows the prevalence of each of these symptoms and conditions, ranked from the most to the least prevalent. Dysmenorrhea and vaginal discharge were the most common symptoms affecting more than 40% of women, while dyspareunia; a symptom closely related to PID, affected more than a quarter.

**Table 2:** Prevalence of PID symptoms

	Symptom	Prevalence %
1	Dysmenorrhea	45.2
2	Vaginal discharge	44.6
3	Miscarriage	42.9
4	Menorrhagia	29.8
5	Dyspareunia	26.8
6	Urinary symptoms	26.2
7	Smelly urine	23.8
8	Chronic pelvic pain	13.7
9	Pelvic heaviness	13.1
10	Preterm labour	12.5
11	Infertility	7.7

A score for PID symptoms was then computed for each woman, giving her an overall PID Symptom Score between (0–11). The mean of the PID Symptom Score was 3.3 (SD = 2.3), ranging from 0 to 11, and was tested against a variety of independent factors, using the independent-sample t-test. As shown in Table 3, the independent factors were found to be significantly associated with the PID Symptom Score were: the use of contraceptives (t = − 1.560, p = 0.044), E&C procedures (t = − 5.392, p = 0.000), hysteroscopy (t = − 2.200, p = 0.029), D&C procedures (t = − 2.669, p = 0.008), and HSG (t = − 2.552, p = 0.011). On the other hand, having CS, IUCD, IVF, or IUI were not found significantly associated with the PID Symptom Score.

**Table 3:** Independent factors associated with PID symptom score

Independent factor	T	*P*
CS	0.723	.471
Use of contraceptives	1.560	.044*
IUCD	− .037	.970
E & C	− 5.392	.000*
D & C	− 2.669	.008*
IVF	0.119	.906
IUI	0.897	.602
HSG	− 2.552	.011*
Hysteroscopy	2.200	.029*

^*^Statistically significant (p < .05)

### Determinants of PID symptoms

Multiple linear regression was used to establish the social determinants of PID symptoms among Jordanian women. PID Symptom Score is a dependent variable, and the Parity, Use of Contraceptives, E&C, D&C, HSG, and Hysteroscopy are the independent predictors. ANOVA Regression F of 6.043 (p = 0.000) was significant for these variables. The model’s R = 0.576 and R^2^ = 0.332 indicate that the combination of these six independent predictors (Para, Use of Contraceptives, E&C, D&C, HSG, and Hysteroscopy) can explain approximately 33% of variance in PID symptoms among Jordanian women. Among the independent predictors, only E&C was found to have statistically significant association with the PID Symptom Score (Beta = 0.0361, t = 4.731, p = -0.000). The remaining predictors (Para, Use of Contraceptives, D&C, HSG and Hysteroscopy) did not associate significantly with PID Symptom Scoring. Table 4 shows the standardized coefficient (Beta), t statistic, and p value for each of the independent predictors of this multiple linear regression model.

**Table 4:** Multiple linear regression model of PID symptom score

Predictor	Beta	t	*p*
Para	0.020	0.245	.807
Use of contraceptives	0.017	0.217	.829
E & C	0.361	4.731	.000
D & C	0.075	0.944	.347
HSG	0.119	1.553	.122
Hysteroscopy	0.077	0.998	.320

Model’s ANOVA regression F = 6.043, p = .000, R = .576, R^2^ = .332

*Statistically significant (p < .05)

## Discussion

Nowadays, smart patient management is becoming increasingly important in health care. Several attempts have been undertaken, globally and in Jordan [22]. One way to be efficient in health care delivery is to understand and address the most important health issues. Pelvic Inflammation Disease is obviously an underdiagnosed disease that silently plagues women, worldwide. The aim of this study is to contribute to the task of understanding PID in Jordan and assess the practices that contribute to its appearance.

We developed a PID symptom scoring system,, ranging from 0 to 11, coupled with a focused questionnaire, that assesses the PDI risk and shows significant association with possible causes of PID. These risk factors include the use of contraception and the insertion of medical instruments in the uterus (e.g. uterine evacuation, hysteroscopic procedures and hysterosalpingograms). Medical instruments might introduce pathogens to the uterus [23], therefore intrauterine contraception devices (IUDs) have been implicated to PID development [24]. Oral/hormonal contraceptive (OC), on the other hand, fail to prevent sexually transmitted infections and thus contribute to a higher PID risk [25], although older reports suggested that OCs could protect against gonorrhoea and reduce PID by 40% [26, 27]. Although condoms are clearly the best protective means to vaginal infection and thus to PID, only 15 (8.9%) of the women in our study reported using it and one third of those (5 cases) used it inconsistently, alternating it to other methods.

The use of condom is a controversial subject, by itself. A recent study in AIDS-infested Nairobi revealed that condom usage is frown upon by both Muslim and Catholic church leaders, as an indirect call for sexual promiscuity [28]. Another study on college students in Canada revealed that female condom carriers are still judged negatively [29], even by other females, suggesting that condom usage still relies on male’s disposition. A recent study in Jordan about the contraceptions’use, as reported by women, showed that 38.3% did not use any kind of contraception and only 42.3% were using some medically approved method (pills, condoms). Some were also relying in non-approved methods (withdrawal and cycle timing). The majority of those using approved methods were educated, living in the urban areas, with those living in the south having the lower implementation rates. The Ministry of Health in Jordan has started rigorous family planning programs over the past 2 decades to reduce the fertility rate and to give access to them in rural areas, but no specialised plan to promote condoms over pills has been implemented to date [30].

Despite the finding that pregnant women, in Amman Jordan, exhibit a high incidence of Group B Streptococcus colonization [31], previous reports on Chlamydia and Gonorrhoea prevalence in the general Jordanian population, found it was low [17], inferring a similarly low PID prevalence. This is also depicted by the fact that none of our participants was ever previously diagnosed with PID, reflecting the local physicians’ widespread notion that PID does not happen in sexually conservative societies. However, our study reveals that the PID-related condition and the frequency of PID symptoms are surprisingly high in our community, although these symptoms could be attributed to other pathologies. Nevertheless, PID is strongly suspected to the symptoms described, especially since frequent alternative aetiologies, such as endometriosis and pelvic pathologies, are not prevalent in the Jordan [30].

Our data also suggest that the women experienced mild chronic symptoms, suggesting that chronic or subclinical PID is more prevalent, while acute PID is rare, which is consistent with the literature. However, the distribution of symptoms in relation to age and parity is different, because the literature states that younger women have higher PID prevalence [31], while in our study the symptoms are equally distributed among ages and parities, with no significant statistical. This observation clearly supports the notion of a different aetiology and pathophysiology. Our data predict that women with higher parity, who used contraceptives, underwent E&C, D&C, HSG, or Hysteroscopy are expected to score higher in the PID Symptom Score.

It is worth noting that women came to accept the disease discomfort as part of their life, not actually seeking treatment. This is a rather common pattern for this disease and largely contributes to its underreporting, and possibly could reflect cultural beliefs. A report about foreign women in Sweden stated that the highest risks of PID were found among women from southern Europe, Eritrea/Ethiopia/Somalia, and other African countries, although Sweden offers publicly financed health care to all [32]. A previous report, in Jordan, showed that women are not adequately aware of their healthcare. Almost half (47.2%) of health care workers did not know that a Pap test was freely available to them and only 26% of them knew the existence of an HPV-vaccine [33].

The main limitation of our study is the small number of participants and the centralised location which gives us only a glimpse of the national picture. Therefore, we need to expand our research to more centres across the country and poll more women with different societal backgrounds. We also have to include those in our statistical evaluation, if we are going to make a viable proposal to the Ministry of Health for nationwide use of this system.

In summary, PID is a potential health issue in Jordan and sexual transmitted diseases do not seem to be the main culprit, given the conservative society structure. Therefore, alternative aetiologies have to be thoroughly investigated and pathogen prevalence studies to be conducted. Our newly developed PID scoring system can provide useful insights and highlight high risk behaviours.

## Conclusion

Chronic/subacute PID is probably quite common in our population and has a wide age of distribution. Women who have high parity, use contraception, and those who underwent uterine instrumentation are at high risk. Though Jordan is a conservative society but PID should be considered whenever symptoms are suggestive.

The small number of participants may hamper the power of the study but reflects, nevertheless, the size of our country. We understand that more rigorous sampling is needed to validate our scoring system and assess more accurately the PID prevalence in Jordan.

## Data Availability

The datasets used and/or analysed during the current study are available from the corresponding author on reasonable request.

## Abbreviations

PID: Pelvic inflammatory disease
C/S: Caesarean section
D&C: Dilation and curettage
IVF: In vitro fertilization
ICSI: Intracytoplasmic sperm injection
HSG: Hysterosalpingography
IUCD: Intrauterine contraceptive device
OC: Oral contraceptive
